# Rapid detection of pecan root-knot nematode, *Meloidogyne partityla*, in laboratory and field conditions using loop-mediated isothermal amplification

**DOI:** 10.1371/journal.pone.0228123

**Published:** 2020-06-18

**Authors:** Sumyya Waliullah, Jessica Bell, Ganpati Jagdale, Tammy Stackhouse, Abolfazl Hajihassani, Timothy Brenneman, Md. Emran Ali

**Affiliations:** 1 Department of Plant Pathology, University of Georgia, Tifton, Georgia, United States of America; 2 Department of Plant Pathology, University of Georgia, Athens, Georgia, United States of America; University of Hyogo, JAPAN

## Abstract

*Meloidogyne partityla* is the dominant root-knot nematode (RKN) species parasitizing pecan in Georgia. This species is known to cause a reduction in root growth and a decline in the yields of mature pecan trees. Rapid and accurate diagnosis of this RKN is required to control this nematode disease and reduce losses in pecan production. In this study, a loop-mediated isothermal amplification (LAMP) method was developed for simple, rapid, and on-site detection of *M*. *partityla* in infested plant roots and validated to detect the nematode in laboratory and field conditions. Specific primers were designed based on the sequence distinction of the internal transcribed spacer (ITS)-18S/5.8S ribosomal RNA gene between *M*. *partityla* and other *Meloidogyne* spp. The LAMP detection technique could detect the presence of *M*. *partityla* genomic DNA at a concentration as low as 1 pg, and no cross reactivity was found with DNA from other major RKN species such as *M*. *javanica*, *M*. *incognita* and *M*. *arenaria*, and *M*. *hapla*. We also conducted a traditional morphology-based diagnostic assay and conventional polymerase chain reaction (PCR) assay to determine which of these techniques was less time consuming, more sensitive, and convenient to use in the field. The LAMP assay provided more rapid results, amplifying the target nematode species in less than 60 min at 70°C, with results 100 times more sensitive than conventional PCR (~2–3 hrs). Morphology-based, traditional diagnosis was highly time-consuming (2 days) and more laborious than conventional PCR and LAMP assays. These features greatly simplified the operating procedure and made the assay a powerful tool for rapid, on-site detection of pecan RKN, *M*. *partityla*. The developed LAMP assay will facilitate accurate pecan nematode diagnosis in the field and contribute to the management of the pathogen.

## Introduction

Pecan (*Carya illinoinensis*) is an important nut crop in North America. A variety of diseases caused by bacteria, fungi, viruses, and nematodes can attack these trees, and if not properly managed, they can cause economic damage to pecans. Root-knot nematodes (RKNs) of the genus *Meloidogyne* are economically important plant-parasitic nematodes, which cause significant damage to pecan production. Three species of RKNs, *Meloidogyne incognita*, *M*. *arenaria*, and *M*. *partityla* have been reported as pathogenic to pecan [1–3]. Among these species, *M*. *partityla* is the dominant RKN parasitizing pecan which has a greater incidence in the southern United States [2–6]. *M*. *partityla* was originally found infecting pecan in South Africa and was likely introduced into the United States during the importation of infected pecan seedlings [7]. Identification of RKN species is essential for the design of a sustainable management option such as resistant cultivars. So that breeders can select for resistance to targeted RKN species/populations. Specific and rapid identification of RKN is crucial not only for genetic and biological variability studies but also to avoid the spread of quarantine pathogens. Members of the genus *Meloidogyne* are extremely adaptable and are highly diverse, genetically, morphologically, and biologically, and their identification using classical methods has proven difficult and time-consuming. Due to the complexity of RKN diagnosis at the species level, it is difficult to conduct extensive surveys, which are required for determining the pecan RKN distribution. Therefore, a quick diagnosis approach will be helpful to know the latest status of the pecan RKNs in the southern United States.

Detection of the RKNs based on the morphological characteristics is an extremely difficult diagnostic task due to the small, microscopic size of RKN and the difficulty to distinguish key diagnostic characters/features under a conventional light microscope [8–10]. In addition, the highly conserved and identical morphology in *Meloidogyne* spp. make it even harder for their accurate identification [11]. Besides morphological characteristics, RKNs can also be identified based on isozyme patterns, and host plant response to infection, which is also challenging for the identification at the species level [8, 12]. Furthermore, extensive knowledge of nematode taxonomy is required for proper RKN identification. Nucleic acid-based molecular methods mostly rely on the polymerase chain reaction (PCR), such as species-specific PCR, multiplex PCR, real-time PCR (qPCR), and PCR-fragment length polymorphism (PCR-RFLP) [13, 14]. Furthermore, random amplified polymorphic DNA (RAPD), high throughput genome sequencing, DNA microarrays, and satellite DNA probes-based hybridization techniques have also been reported to distinguish nematode species [11, 15, 16]. However, all of these molecular methods are time-consuming and require access to sophisticated and bulky laboratory equipment. In particular, for *M*. *partityla* identification, a PCR based assay targeting the fragment between mitochondrial COII gene and the large (16S) rRNA gene of mitochondrial DNA regions is widely used. It is a fast and sensitive tool compared to existing traditional methods for identification [13]. However, this PCR based diagnostic procedure still needs a few hours (~2–3 hrs) and expensive laboratory instrumentation. Due to these drawbacks, a detection method that is not only quick but also simple and economical is clearly needed.

Loop-mediated isothermal amplification (LAMP) is a novel technique that can overcome many of the limitations of traditional microscopy and molecular PCR based diagnostic assays [17–20]. It has been reported that LAMP sensitivity can be 100 to 1,000 times higher than conventional methods and that it can easily detect below 1pg/μl or lower concentrations [21]. This method costs less time per sample and is simpler to perform than other detection methods. It can be carried out rapidly (often in 30 min) with minimal equipment (a water bath or isothermal heat block) which is highly applicable for the onsite diagnosis of pathogens [22, 23]. Recently LAMP technique has been used for identifying different species of RKNs including *M*. *enterolobii*, *M*. *incognita*, *M*. *arenaria*, *M*. *javanica*, *M*. *hapla*, *M*. *chitwoodi* and *M*. *fallax* [22–25]. However, so far there is no report for using the LAMP technique for the identification of *M*. *partityla*.

Due to the limitations of currently used methods, our objective was to develop a loop-mediated isothermal amplification (LAMP) assay for specific and rapid detection of *M*. *partityla* in pecan under laboratory and field conditions, and to determine the time needed to conduct the procedure.

## Results

### Morphological identification

Based on the morphological characteristics, the tested RKN’s were diagnosed as *M*. *partityla* (S1 Fig). Morphological measurements (mean ± SD, range) of RKN J2 (n = 20) included mean body length (441.56 ± 17.41 μm, 412.50–468.75 μm), maximum body width (15.89 ± 1.22 μm, 14.06–18.75 μm), stylet length (14.76 ± 1.26 μm, 11.71–15.62 μm), tail length (48.20 ± 2.45 μm, 43.75–53.13 μm), and hyaline region of the tail (15.82 ± 0.94 μm, 14.06–18.75 μm).

### Optimization of LAMP reaction

**Fig 1:**
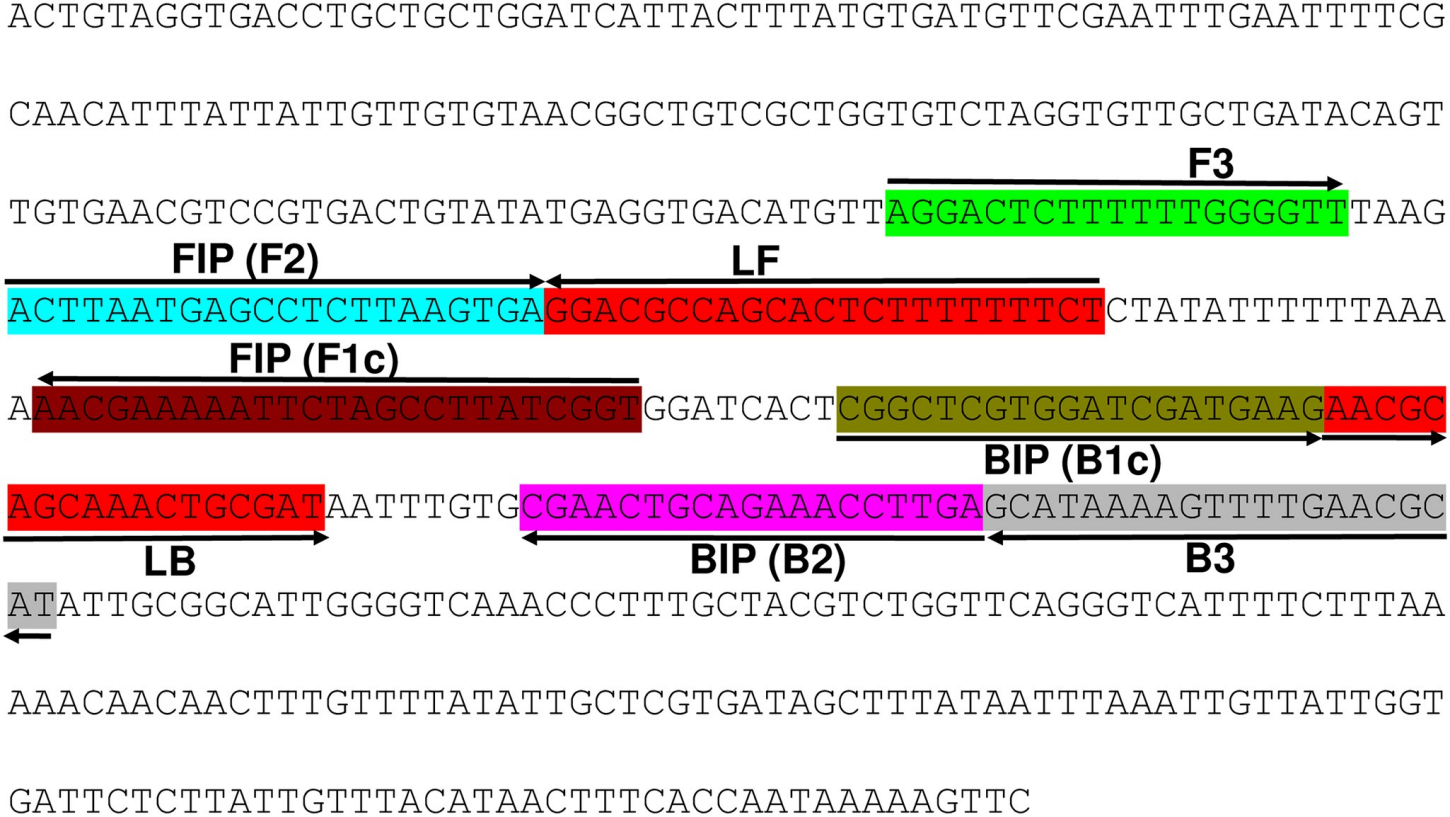
Location and partial sequence of loop-mediated isothermal amplification (LAMP) primer set targeting *Meloidogyne partityla* specific gene Internal Transcribed Spacer (ITS)-18S/5.8S ribosomal RNA. Primer locations for LAMP assay (F3, B3, FIP [F1c-F2], and BIP [B1c-B2]). FIP is a hybrid primer consisting of the F1c sequence and the F2 sequence, BIP is a hybrid primer consisting of the B1c sequence and the B2 sequence. Arrows indicate the extension direction.

**Fig 2:**
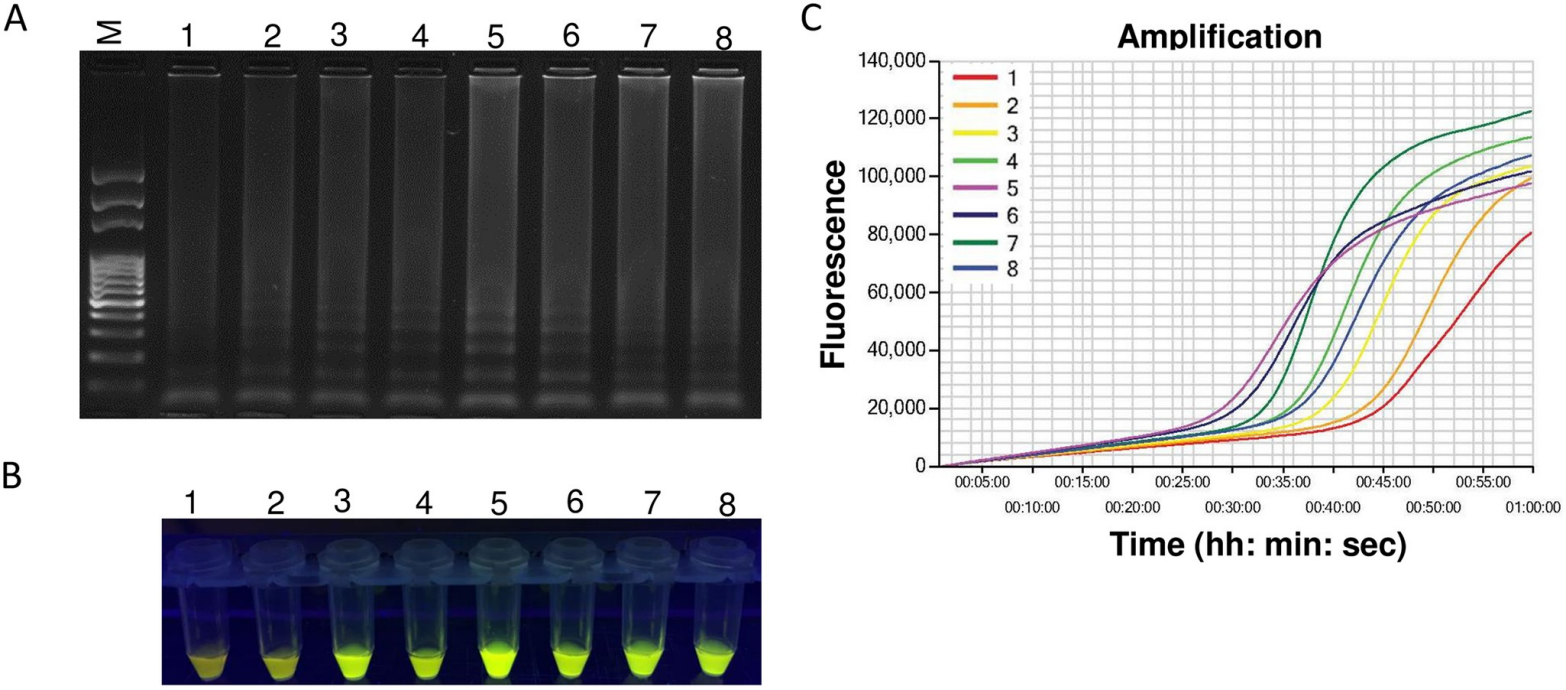
Optimization of incubation temperature for LAMP reaction. Assessment was based on (A) agarose gel electrophoresis of the LAMP products (B) visualization after the addition of SYBR Green I nucleic acid stain into the reaction tubes under UV light exposure, fluorescent green color represents positive amplification; (C) real-time amplification by Genie^®^ III. Here, 1: 66°C, 2: 67°C, 3: 68°C, 4: 69°C, 5: 70°C, 6: 71°C, 7: 72°C and 8: 73°C. Lane M: 100 bp DNA ladder.

The LAMP assay was optimized using different LAMP primer concentrations, incubation temperatures, and duration using DNA extracted from *M*. *partityla*. The final LAMP primer mix (Fig 1, Table 1) used in this study contained: 0.2μM of each F3 and B3 primers, 0.8μM of each Loop F and Loop B primers, and 1.6μM of each FIP and BIP primers (data not shown). For incubation temperature and time, the best reaction performance was found at 70°C for 60 minutes in 0.2-ml micro-tubes in a thermocycler or Genie^®^ III (Fig 2). After gradient amplifications for the optimization of temperature, the LAMP products presented characteristic ladder-shaped band pattern from 1000 bp to less than 100 bp when separated using 1% agarose gel electrophoresis (Fig 2A), where the brightest band was obtained from 70°C (Fig 2, S1 Table). The finding was supported by adding SYBR^™^ Green 1 nucleic acid gel stain (Invitrogen, Carlsbad, CA). After amplification, positive reaction at 70°C reactions appeared as the brightest fluorescent green under UV light by adding SYBR Green I fluorescence dye (Fig 2B). Along with the gel electrophoresis and SYBR^™^ Green I nucleic acid gel stain, less time was taken for amplification at that particular temperature during amplification using Genie^®^ III (S1 Table). In addition to these detection methods, an amplified LAMP reaction was also confirmed by viewing the real-time graphs on Genie^®^ III (Fig 2C).

**Table 1:** Primers used in this study.

Purpose	Primer name	Primer sequence (5'-3')	Target gene	Source
PCR	C2F3	GGTCAATGTTCAGAAATTTGTGG	Mitrocondian DNA	[13]
identification	1108	TACCTTTGACCAATCACGCT		
LAMP assay	Mp-F3	AGGACTCTTTTTTGGGGTT	Ribosomal DNA	This study
	Mp-FIP	ACCGATAAGGCTAGAATTTTTCGTT*TTTT*ACTTAATGAGCCTCTTAAGTGA		
	Mp-LF	AAAAAGAGTGCTGGCGTCC		
	Mp-B3	ATGCGTTCAAAACTTTTATGC		
	Mp-BIP	CGGCTCGTGGATCGATGAAG*TTTT*TCAAGGTTTCTGCAGTTCG		
	Mp-LB	AACGCAGCAAACTGCGAT		

### Detection of *M*. *partityla* in laboratory condition

**Fig 3:**
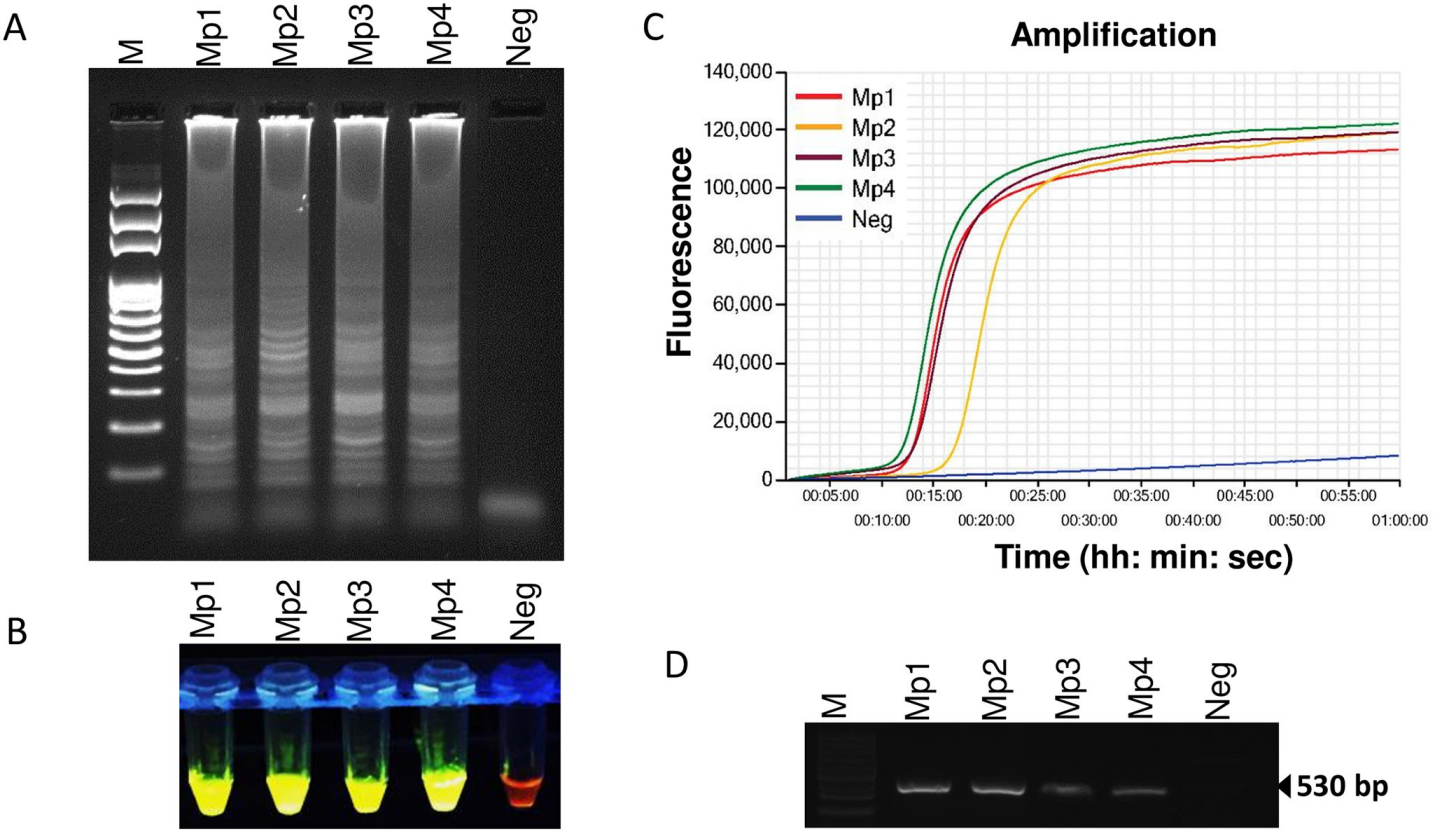
Loop-mediated isothermal amplification of DNA from four pure culture *Meloidogyne partityla*. Assessment was based on (A) agarose gel electrophoresis of the LAMP products; (B) visualization after addition of SYBR Green I nucleic acid stain into the reaction tubes under UV light exposure, fluorescent green color represents positive amplification; (C) real-time amplification by Genie^®^ III; (D) PCR amplification using primer pair C2F3/1108. Lane M: 100 bp DNA ladder; Mp1, Mp2, Mp3, and Mp4: four different isolates of *Meloidogyne partityla*; Neg: negative control.

To demonstrate the applicability of the LAMP assay for the detection of *M*. *partityla*, the method was evaluated using DNA collected from an individual female originated from infested pecan root galls which were also previously identified as *M*. *partityla* by PCR reactions and morphological studies (Fig 3D, S1 Fig). All four isolates of *M*. *partityla* manifested positive amplification of LAMP with gel electrophoresis (Fig 3A), SYBR^™^ Green I nucleic acid gel stain (Fig 3B), and Genie^®^ III (Fig 3C). Considering all three different laboratory detection methods, traditional morphology-based detection system required a longer time (about 2 days) than other molecular methods i.e. PCR and LAMP.

### Specificity of LAMP assay

**Fig 4:**
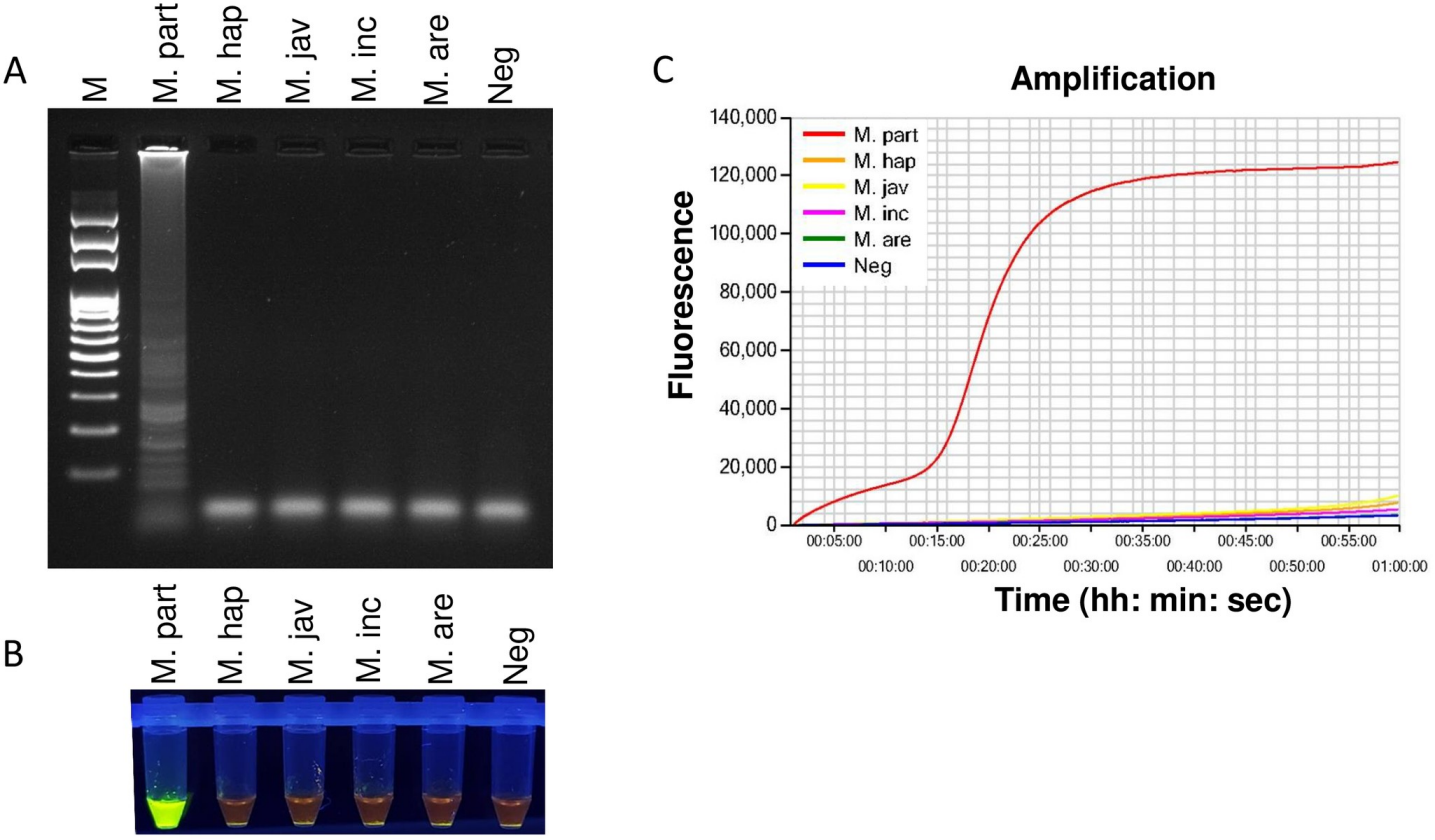
Specificity determination of LAMP assay using DNA from pure cultures of five different *Meloidogyne* spp. Assessment was based on (A) agarose gel electrophoresis of the LAMP products; (B) visualization after addition of SYBR^™^ Green I nucleic acid stain into the reaction tubes under UV light exposure, fluorescent green color represents positive amplification; (C) real-time amplification by Genie III. Lane M: 100 bp DNA ladder; M. part: *Meloidogyne partityla*; M. hap: *Meloidogyne hapla*; M. jav: *Meloidogyne javanica*; M. inc: *Meloidogyne incognita*; M. are: *Meloidogyne arenaria*; Neg: negative control.

Specificity of the designed primers was assessed using five *Meloidogyne* spp. Only *M*. *partityla* resulted in positive amplification, while no amplification was observed for *M*. *hapla*, *M*. *javanica*, *M*. *incognita*, and *M*. *arenaria* by the LAMP assay (Fig 4). Results were confirmed using three different detection strategies including agarose gel doc image analysis (Fig 4A), SYBR^™^ green-based UV image (Fig 4B), Genie III amplification curve analysis (Fig 4C). All three detection strategies showed a positive reaction only from *M*. *partityla*, but not from other nematode species (Fig 4). The results indicated that the LAMP assay could distinguish *M*. *partityla*, from closely related *Meloidogyne* spp. The nucleotide differences between *M*. *partityla* and other closely related *Meloidogyne* spp. within targeted ITS region also support the idea of specific detection of *M*. *partityla* (S2 Fig).

### Sensitivity comparison of LAMP with conventional PCR

**Fig 5:**
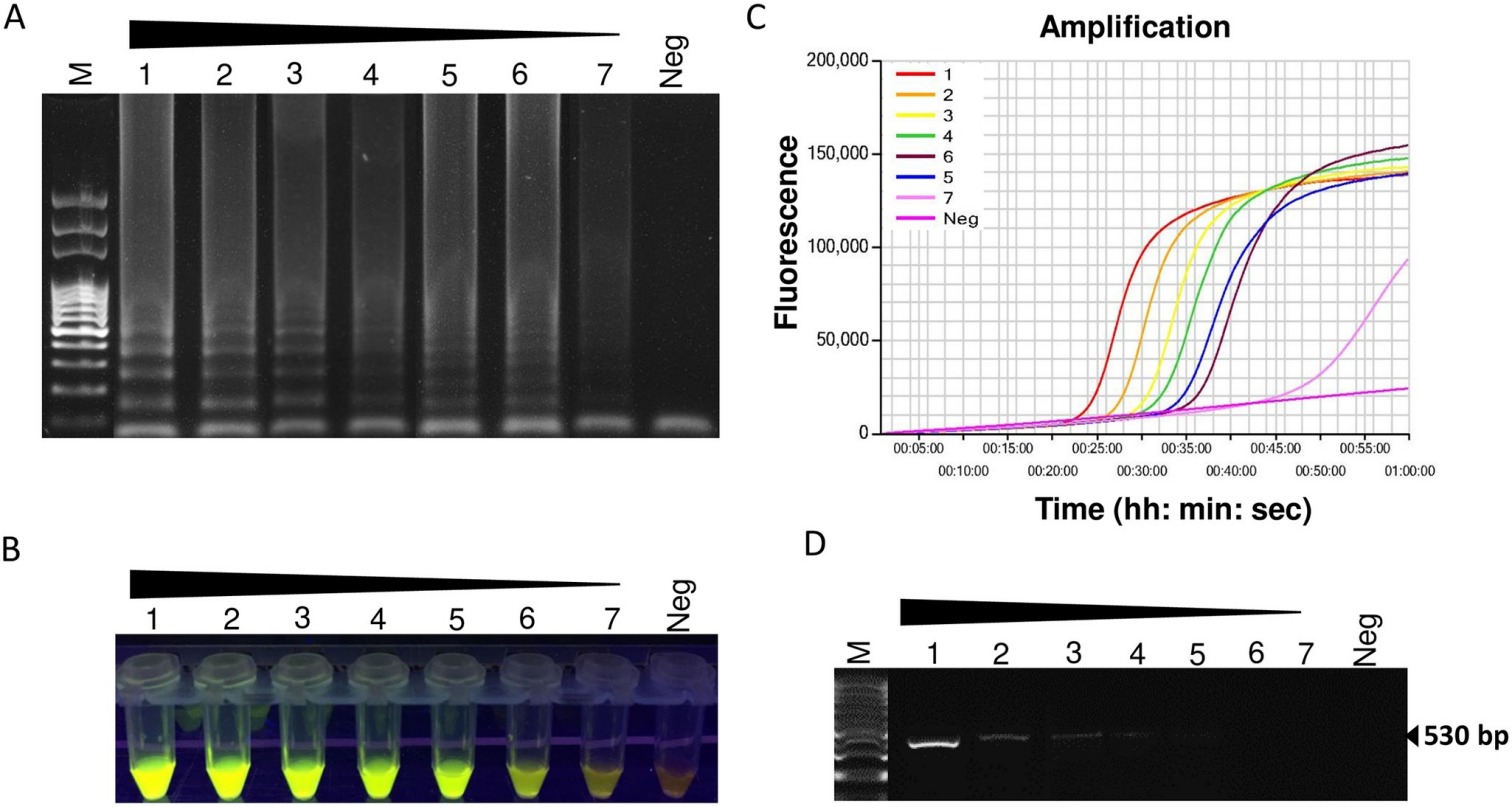
Sensitivity of LAMP assay using 10-fold serially diluted DNA extracted from the pure culture of *Meloidogyne partityla*. Assessment was based on (A) agarose gel electrophoresis of the LAMP products (B) visualization after addition of SYBR Green I nucleic acid stain into the reaction tubes under UV light exposure, fluorescent green color represents positive amplification; (C) real-time amplification by Genie^®^ III; (D) PCR amplification using primer pair C2F3/1108. Lane M: 100 bp DNA ladder; numbers 1 to 7: 10-fold serial dilution of *M*. *partityla DNA* from 100 ng/μl to 10−4 ng/μl; Neg: negative control.

Ten-fold serial dilutions of purified genomic DNA from a single female nematode (n = 3) were used to evaluate the sensitivity of the LAMP method. Positive amplifications were viewed with gel electrophoresis (Fig 5A), SYBR^™^ Green I nucleic acid stain based UV image of the reaction tubes (Fig 5B), and Genie^®^ III real-time graphs (Fig 5C) until 0.1 pg per reaction, indicating that the detection limit was 1 pg of genomic DNA. For conventional PCR, the expected fragment was amplified when the template DNA was diluted up to 10 pg. Thus, for purified DNA, LAMP was at least 100-fold more sensitive than conventional PCR (Fig 5D). No amplification was observed in the no-template control.

### Onsite detection in infested plant root galls using LAMP assay

**Fig 6:**
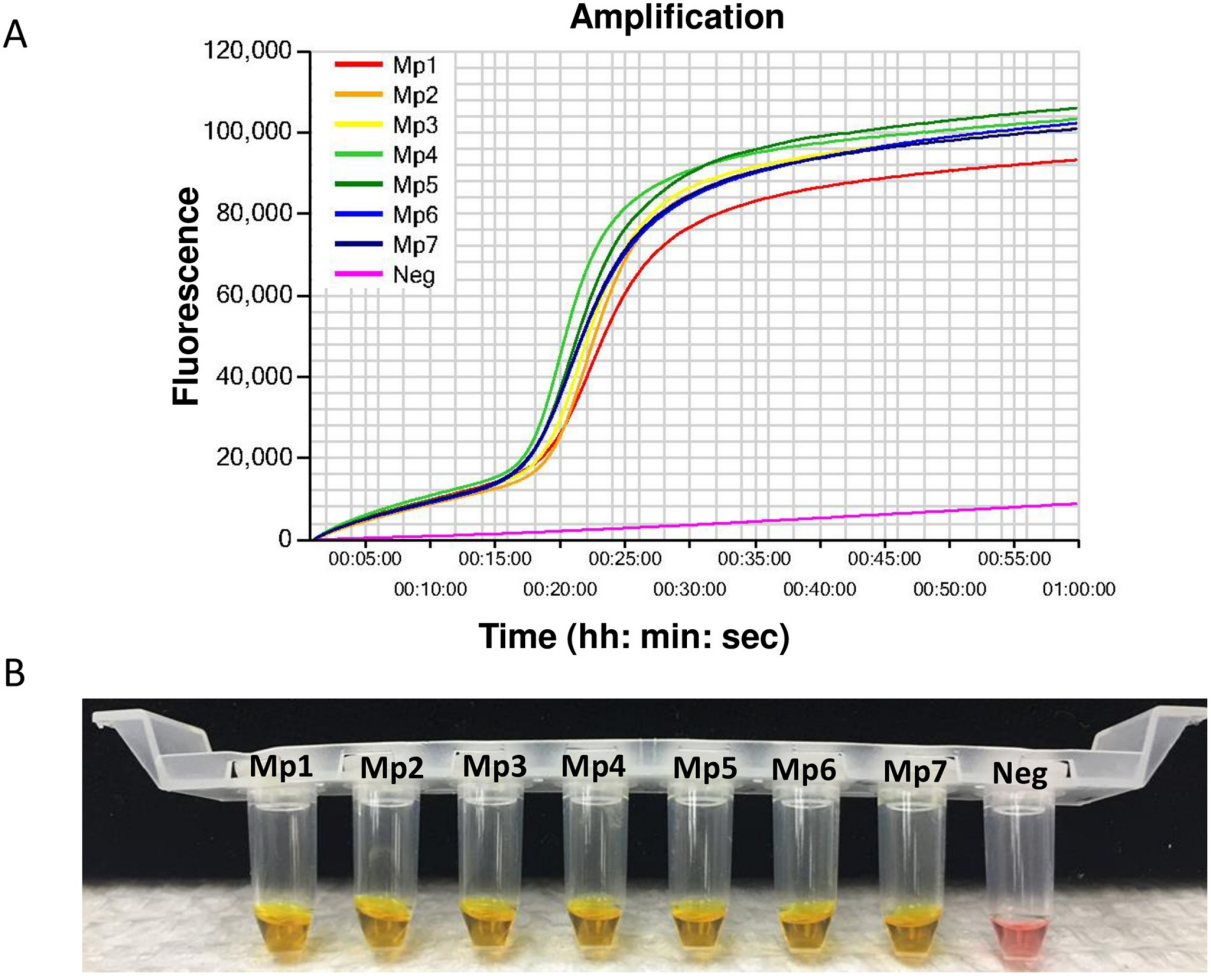
LAMP detection of *Meloidogyne partityla* from seven infected pecan root gall samples. Detection was based on (A) real-time amplification by Genie^®^ III; (B) visualization using WarmStart colorimetric dye to determine positive reaction by the naked eye for the field detection. Yellow color represents positive amplification while no amplified product remained pink. Mp1 to Mp7: seven different field isolates of *Meloidogyne partityla*; Neg: negative control.

Seven infested root gall samples were collected from the UGA Ponder research farm to evaluate the applicability of this assay for real world examples (S3 Fig). All samples showed positive results in the LAMP reaction confirming as *M*. *partityla* (Fig 6A and 6B).

## Discussion

The specific and quick diagnosis of RKNs is required for disease prediction and adequate management control strategy selection. Traditionally, *M*. *partityla* was diagnosed based on morphological observation, and PCR based molecular methods which are time-consuming processes and require highly trained personnel and laboratory instruments [26, 27]. The LAMP assay is an effective tool for plant-parasitic nematode identification because of its capability of DNA amplification at isothermal conditions with high sensitivity and efficiency [18, 22–25]. In this report, we present a novel method to detect the pecan RKN, *M*. *partityla*. The identification can be completed in an onsite setting using a handheld magnifier and portable incubator. The amplified products can be detected visually by the naked eye and/or with the Genie^®^ III instrument to view the amplification graph on the screen within 2 hours. Several LAMP assays have been developed to detect common *Meloidogyne* spp. [22–25]. Some of these LAMP sets are very specific to a certain RKN species, and in some cases, a single LAMP primer set can also be used for detecting double or multiple RKNs simultaneously [23, 25]. In 2019, Zhang et al. developed a LAMP primer set that can detect two potato nematodes, *M*. *chitwoodi*, and *M*. *fallax* [25]. A universal RKN-LAMP was also reported that can identify the four common *Meloidogyne* spp.: *M*. *incognita*, *M*. *arenaria*, *M*. *javanica* and *M*. *hapla* [23]. Here we designed LAMP primers targeting the conserved ITS region of *M*. *partityla* using the PrimerExplorer V5 software (Fig 1, Table 1). The targeted ITS region contains significant nucleotide differences between *M*. *partityla* and other closely related *Meloidogyne* spp. which reduces the risk of misdetection (S2 Fig). Based on test results, this newly designed LAMP assay showed high specificity which only yields positive results with *M*. *partityla* and provides us with an essential tool to identify *M*. *partityla* correctly (Figs 3 and 4).

Additionally, we present evidence that LAMP is faster than traditional morphology-based microscopic methods. The entire morphological diagnosis process took about 2 days which is very consistent with other reported observational methods of RKN diagnosis that mostly rely on the distinct morphological and anatomical characteristics of second-stage juveniles, adult males, as well as perineal patterns of adult females [10, 28–30]. Our results based on the morphological characteristics of J2s in this study were in agreement with those reported for *M*. *partityla* [26, 27] and confirm the reliability of the LAMP technique for the rapid identification of *M*. *partityla*. PCR based traditional molecular techniques have also been frequently used to detect *M*. *partityla* by designing primers on the ribosomal intergenic spacer (rDNA). However, Our results showed these techniques are less sensitive than LAMP as seen by LAMP detection of 1 pg pure genomic DNA which was 100 times lower than that of PCR-based detection methods (Fig 5). This finding was supported by previously published reports on LAMP for other pathogens [21, 24, 31].

LAMP has recently been used as an on-site diagnosis technique for rapid and sensitive detection of multiple pathogens as the assay only requires a single temperature rather than a thermal cycler [32, 33]. In this study, we developed a LAMP-based quick detection protocol of *M*. *partityla* which can be done in on-site settings. This method can be run in the field using a colorimetric dye for visual confirmation or using a real-time amplification machine for results in less than one hour [34, 35]. In this study, direct extraction of DNA from a single female isolated from root galls greatly improved the efficiency of the Extract-N-Amp^™^ Tissue PCR Kit. This extraction process is rapid and only requires a portable incubator. Our onsite study demonstrated that the LAMP assay which combined a handheld magnifier and an incubator was fully applicable to plant nematode detection (Fig 6, S3 Fig). To the best of our knowledge, this is the first published demonstration of detecting plant nematodes using the LAMP assay in a field setting. Combined with similar observations from previous reports [22, 36], these results showed that this assay has a great simplicity and can overcome various types of limitations for using prepared tissue suspension, including the need for a laboratory setting. This also demonstrates the applicability of LAMP as a sensitive assay for determining species level at field condition. Field samples can be collected and DNA immediately extracted using the Extract-N-Amp^™^ Tissue PCR Kit. Those samples can then be tested for the presence of *M*. *partityla* using the user-friendly, efficient LAMP method, all in the span of 60 minutes.

In summary, the portable LAMP assay described here was shown to be very reliable for the rapid detection of *M*. *partityla*. It is simple to operate, accurately differentiates among *Meloidogyne* spp., provides results quickly, and does not require specialized equipment in comparison with the traditional morphology-based detection techniques and PCR based molecular methods. The LAMP assay will be invaluable to those working with this nematode, and will greatly increase our ability to monitor and manage *M*. *partityla* in pecan.

## Materials and methods

### Source of RKNs

*Meloidogyne* spp. and all other plant nematodes used in this study were collected from the Nematode Laboratories, University of Georgia. All *Meloidogyne* spp. were purified from a single egg-mass. *M*. *partityla* was collected from infested roots of a pecan tree which was previously confirmed for the presence of only one RNK; *M*. *partityla*. Egg-masses of each of *M*. *hapla*, *M*. *javanica*, *M*. *incognita*, and *M*. *arenaria* were collected from infested roots of tomato plants maintained in the greenhouse in Athens, GA.

### Morphological assay

For morphological identification, eggs of *M*. *partityla* were collected from RKN infested pecan roots following the method of Hussey and Barker, 1973 [37], and the species identification was performed following the protocol as mentioned in S1 Fig. In brief, after collecting adult females of pecan RKN, perineal patterns were prepared for the species identification. The collected eggs were also transferred to a petri dish containing water and allowed to hatch into second-stage juveniles (J2) for 7 days at room temperature (25°C). For morphological measurements, 20 J2 were hand-picked and temporary water mounts were prepared. Morphological measurements of each J2 were recorded with a Leica DME compound research microscope using an eyepiece micrometer at 400x magnification.

### Laboratory DNA extraction

Two different methods were used to extract genomic DNA from the root knot nematode. For the laboratory optimization step, genomic DNA was extracted from an adult female of each RKN species using the QIAGEN DNeasy^®^ Blood & Tissue Kit (Qiagen, Valencia, CA) with some modifications. Briefly, the adult female was punctured with a dissecting needle 6–8 times, and added in 90 μl ATL buffer and incubated overnight at 56°C after adding 20 μl of proteinase K. The female was then vortexed for 15 sec before adding 100 μl Buffer AL and 100 μl ethanol (96%). The mixture was placed in a DNeasy mini spin column and centrifuged at 8000 rpm for 1 min and flow-through was discarded. With a new receiving tube, the centrifugation procedure was repeated adding 200 μl Buffer AW1 and then 200 μl Buffer AW2 to dry the DNeasy membrane at 14000 rpm for 3 min, discard the flow-through each time. The DNeasy Mini spin column was placed onto a 1.5 ml microcentrifuge tube and 25 μl Buffer AE was directly pipetted onto the DNeasy membrane to elute the DNA. Then the DNA was allowed to stand for 5 min before being centrifuged at 6000 rpm for 1 min. The elution step was repeated one more time with an additional 25 μl Buffer AE. The resulting DNA was stored at –20°C for subsequent experiments.

### PCR amplification

The forward primer C2F3 and the reverse primer 1108 were used to amplify the fragment between the mitochondrial COII gene and the large (16S) rRNA gene [13]. The amplification was carried out under the following cycling conditions: 94°C for 5 min, then 35 PCR cycles of 94°C for 30 seconds, 55°C for 30 seconds, 72°C for 30 seconds and final incubation at 72°C for 10 min. PCR products were analyzed by electrophoresis on a 1% agarose gel and visualized in a UV gel doc machine. This primer (C2F3/1108) was used to detect the sensitivity of traditional PCR. Forward and reverse primer pairs are listed in Table 1.

### Design of LAMP primers

LAMP primers were designed based on the sequence obtained from GenBank (accession number KR047556.1) of the internal transcribed spacer (ITS) region of the ribosomal DNA using PrimerExplorer V5 software (Eiken Chemical Co., Tokyo, Japan). Six primers were constructed: two outer primers (F3 and B3), a forward inner primer (FIP), a backward inner primer (BIP), a loop forward primer (LF), and a loop backward primer (LB). FIP comprised the F1c sequence complementary to the F1 and F2 sequence. BIP consisted of the B1c sequence complementary to the B1 and B2 sequences (Fig 1, Table 1). To check if the primer binding sites of the targeted region contained nucleotide differences between *M*. *partityla* and other *Meloidogyne* spp, other *Meloidogyne* spp sequences were also obtained from GenBank and used for comparison with *M*. *partityla* sequence (S2 Fig). The corresponding GenBank accession numbers for the *Meloidogyne* spp are *Meloidogyne javanica*: accession number AY438555.1, *Meloidogyne arenaria*: accession number AF387092.1, *Meloidogyne hapla*: accession number JX024147.1, *Meloidogyne enterolobii*: accession number KM046989.1 and *Meloidogyne incognita*: accession number JQ405212.1. The selected sequences were imported into Geneious v10.1.2 (Biomatters Ltd., Auckland, New Zealand) for sequence alignment using the Align/Assemble > Pairwise/Multiple Align function using “Geneious Alignment” option with default settings (S2 Fig). The alignment was generated using multiple alignment algorithms and then curated by hand.

### Optimization of the LAMP reaction

The LAMP reaction was performed using LavaLAMP^™^ DNA Master Mix (Lucigen, WI, USA) according to the manufacturer’s instructions. Each reaction contained 12.5 μl 2X master mix, 2.5 μl of primer mix, 1 μl DNA samples and the rest were filled with DNase/RNase free PCR certified water (TEKNOVA, Hollister, USA) to a final volume of 25 μl. The LAMP assay was optimized using different LAMP primer concentrations [F3, B3 (0.1–0.5 μM each); LF, LB (0.5–1.0 μM each) and FIP, BIP (0.8–2.4 μM each)], durations (30–60 min), and temperatures (65–73°C). Optimization of temperature was performed in the Genie^®^ III real-time instrument by determining the value of two parameters: amplification time (Tiamp) and amplicon annealing temperature (Ta) (S1 Table). The temperature with the least amplification time (Tiamp) and the highest peak value of the melting curves (Ta) was considered as the optimum temperature for the assay. After optimization, all reactions were performed in 0.2-ml micro-tubes in a thermocycler (or Genie^®^ III) set to 70°C for 60 minutes and terminated by incubating at 4°C for 5 minutes or Genie^®^ III amplification, the mixture was preheated at 90°C for 3 min, amplified at 70°C for 60 min, and then terminated at a range from 98°C to 80°C, with a decline rate of 0.05°C per second. Each run contained a positive control of pure culture DNA and negative control of PCR grade water without a template.

### Analysis of LAMP products in laboratory

The LAMP amplification results were detected with three different methods in a laboratory setting: 1. on 1% TBE agarose gel stained with GelGreen^®^ Nucleic Acid Stain (Biotium, Fremont, CA) for visual observation; 2. with SYBR^™^ Green 1 nucleic acid gel stain (Invitrogen, Carlsbad, CA) for observation under UV light; and 3. using Genie^®^ III (OptiGene, Horsham, WS, UK) instrument by obtaining the amplification curves and analyzing data using Genie Explorer software (OptiGene, Horsham, WS, UK). All reactions were repeated at least three times.

### Sensitivity comparison of LAMP to conventional PCR

To determine the sensitivity of the LAMP assay, extracted DNA concentration was measured using a NanoDrop Lite (Thermo Scientific) instrument, and a tenfold serial dilution from the extracted DNA was done from 100 ng/μl down to a concentration of 10^−5^ ng/μl and subjected to the LAMP assay and the conventional PCR. Sensitivity tests were repeated three times.

### Specificity of the LAMP assay

Genomic DNA isolated from several *Meloidogyne* spp. including *M*. *partityla*, *M*. *hapla*, *M*. *javanica*, *M*. *incognita*, and *M*. *arenaria* was used to verify the specificity of the LAMP assay. Specificity tests were repeated three times.

### On-site detection of *M*. *partityla*

For the onsite quick diagnosis application, suspected pecan root samples were collected from the UGA Ponder research farm pecan orchard in Tift County, GA, washed with distilled water, and examined under a handheld magnifier (10x) glass for the presence of galls/knots (SPI Swiss Precision Instrument Inc., CA, USA). A single adult female was isolated from a gall and transferred into a 1.5 ml microcentrifuge tube, and DNA was extracted at the field using quick Extract-N-Amp^™^ Tissue PCR Kit (Sigma-Aldrich Co. LLC, St. Louis, MO; USA) with some modifications. Briefly, the single female of RKN was punctured with a dissecting needle 6–8 times and added into a tube containing 50 μl of extraction buffer and 12.5 μL of tissue preparation solution, which was mixed by pipetting up and down for several times. After initial incubation at ambient temperature for 10 minutes, the sample was further incubated at 95°C for 3 minutes. Finally, 50 μL of neutralization solution was added to the tube and mixed it by vortexing and 5 μl of extraction product was directly used as a template for LAMP amplification (S3 Fig). WarmStart^®^ Colorimetric LAMP 2X Master Mix (New England Bio Labs Ltd., UK) which utilizes pH sensitive phenol red as an end-point indicator was used for onsite naked eye detection according to manufacturer’s instruction. Detection was carried out by observing the color change of the WarmStart^®^ colorimetric dye in the naked eye for the field detection (S3 Fig). Each reaction mixture was performed in 0.2-ml micro-tubes either set to 70°C for 60 minutes. Each run contained a positive control of pure culture DNA and negative control of PCR grade water without a template. If the Genie^®^ III instrument was used for onsite real-time detection, the amplification graph was observed on the screen to determine the result (S3 Fig). For Genie^®^ III real-time run, LavaLAMP^™^ DNA Master Mix was used according to the protocol described above.

## Supporting information

S1 Table Optimization of temperature of the LAMP reaction using real-time LAMP detection system Genie^®^ III. Selected temperature for LAMP amplification is highlighted with a bold format.
(DOCX)

S1 Fig Layout for morphology based diagnosis of *Meloidogyne partityla*. (PDF)

S2 Fig Comparison of sequences among closely related RKN’s based on rDNA-ITS sequences. Arrows represent the location of the primers. Here MP, *Meloidogyne partityla*, MJ, *Meloidogyne javanica*, MA, *Meloidogyne arenaria*, MH, *Meloidogyne hapla*, ME, *Meloidogyne enterolobi* and MI, *Meloidogyne incognita*.
(PDF)

S3 Fig Layout for onsite diagnosis of *Meloidogyne partityla*. (PDF)

S1 Raw images (PDF)
