# Supplementary material for: Rapid detection of pecan root-knot nematode, Meloidogyne partityla, in laboratory and field conditions using loop-mediated isothermal amplification
Source: PLoS One. 2020 Jun 18;15(6):e0228123. doi: 10.1371/journal.pone.0228123 (PMC7302683; doi:10.1371/journal.pone.0228123)
Supplement: S1 Table — Selected temperature for LAMP amplification is highlighted with a bold format. (DOCX) [file pone.0228123.s001.docx]

**Supplementary Table S1**. Optimization of temperature of the LAMP reaction using real-time LAMP detection system Genie® III. Selected temperature for LAMP amplification is highlighted with a bold format.

| **Tm_amp_ (°C)^a^** | | **Ti_amp_ (Min:S)^b^** | **Ta (°C)^c^** |
| --- | --- | --- | --- |
| 66 | 48.15 | | 84.1 |
| 67 | 47.3 | | 84.2 |
| 68 | 43.15 | | 84.3 |
| 69 | 40.00 | | 84.6 |
| **70** | **33.00** | | **86.4** |
| 71 | 34.30 | | 86.2 |
| 72 | 36.30 | | 85.4 |
| 73 | 40.45 | | 84.1 |

^a^ Amplification temperature in degree Celsius (°C)

^b^ Amplification time in minutes and seconds (Min:S)

^c^ Annealing temperature in degree Celsius (°C)
