# Supplementary figures and images for: Rapid detection of pecan root-knot nematode, Meloidogyne partityla, in laboratory and field conditions using loop-mediated isothermal amplification
Source: PLoS One. 2020 Jun 18;15(6):e0228123. doi: 10.1371/journal.pone.0228123 (PMC7302683; doi:10.1371/journal.pone.0228123)

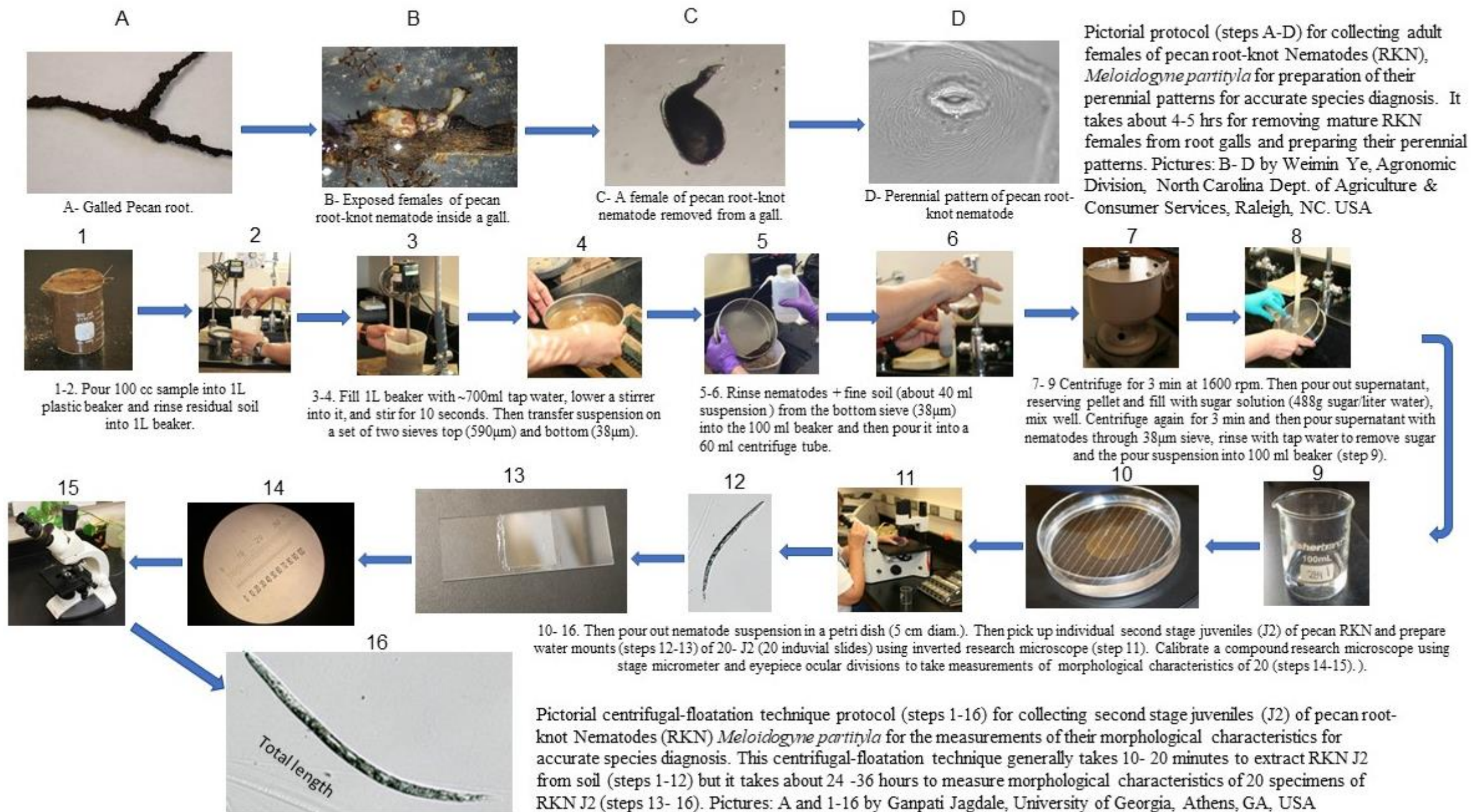

Figure S1 Layout for morphology based diagnosis of *Meloidogyne partityla*

Supplement: S1 Fig — (PDF) [file pone.0228123.s002.pdf]
