# Supplementary material for: Rapid detection of pecan root-knot nematode, Meloidogyne partityla, in laboratory and field conditions using loop-mediated isothermal amplification
Source: PLoS One. 2020 Jun 18;15(6):e0228123. doi: 10.1371/journal.pone.0228123 (PMC7302683; doi:10.1371/journal.pone.0228123)
Supplement: S2 Fig — Arrows represent the location of the primers. Here MP, Meloidogyne partityla, MJ, Meloidogyne javanica, MA, Meloidogyne arenaria, MH, Meloidogyne hapla, ME, Meloidogyne enterolobi and MI, Meloidogyne incognita. (PDF) [file pone.0228123.s003.pdf]

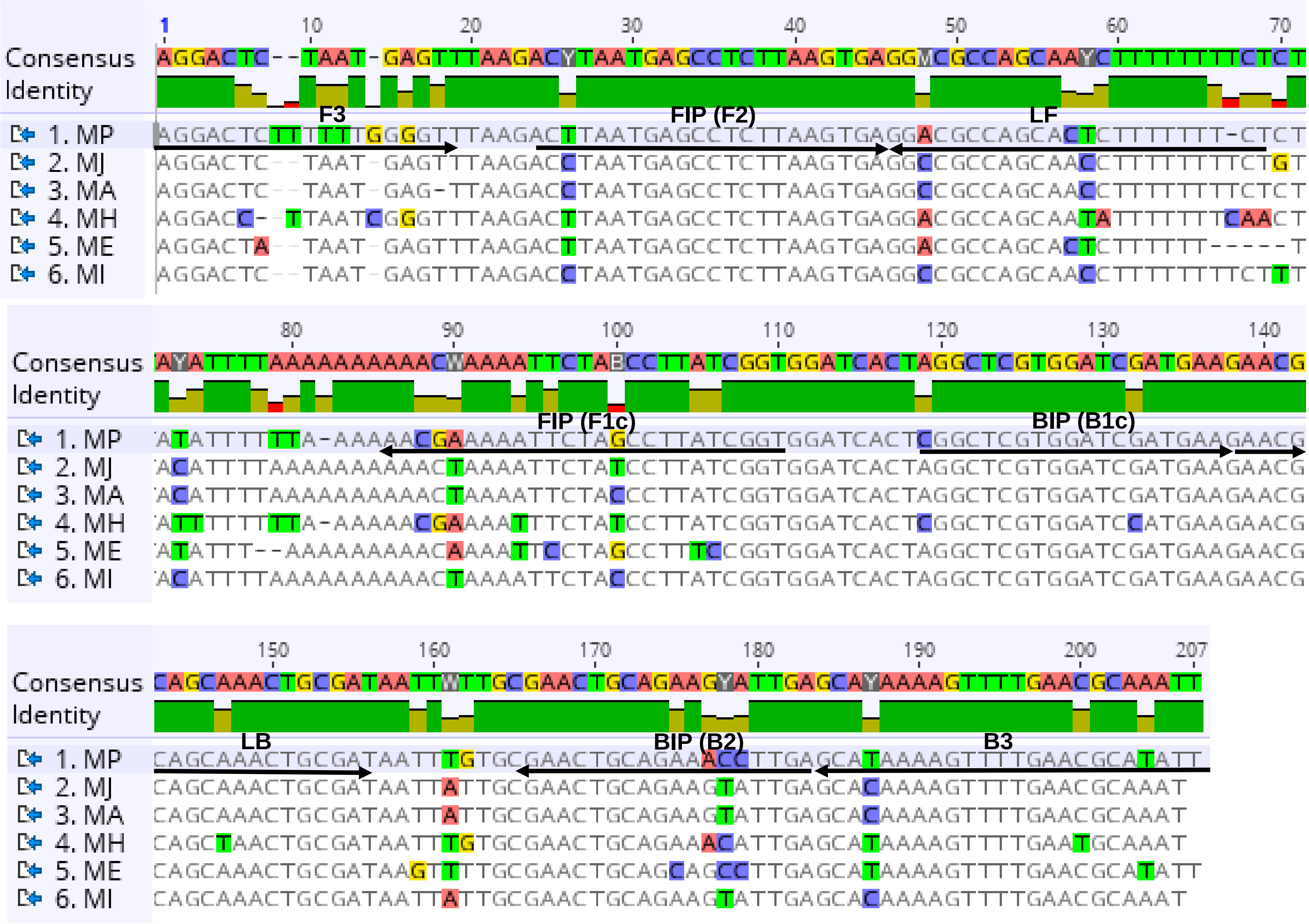

Figure S2 Comparison of sequences among closely related RKN's based on rDNA-ITS sequences. Arrows represent the location of the primers. Here MP, *Meloidogyne partityla*, MJ, *Meloidogyne javanica*, MA, *Meloidogyne arenaria*, MH, *Meloidogyne hapla*, ME, *Meloidogyne enterolobi* and MI, *Meloidogyne incognita*.
