# Supplementary material for: Rapid detection of pecan root-knot nematode, Meloidogyne partityla, in laboratory and field conditions using loop-mediated isothermal amplification
Source: PLoS One. 2020 Jun 18;15(6):e0228123. doi: 10.1371/journal.pone.0228123 (PMC7302683; doi:10.1371/journal.pone.0228123)
Supplement: S3 Fig — (PDF) [file pone.0228123.s004.pdf]

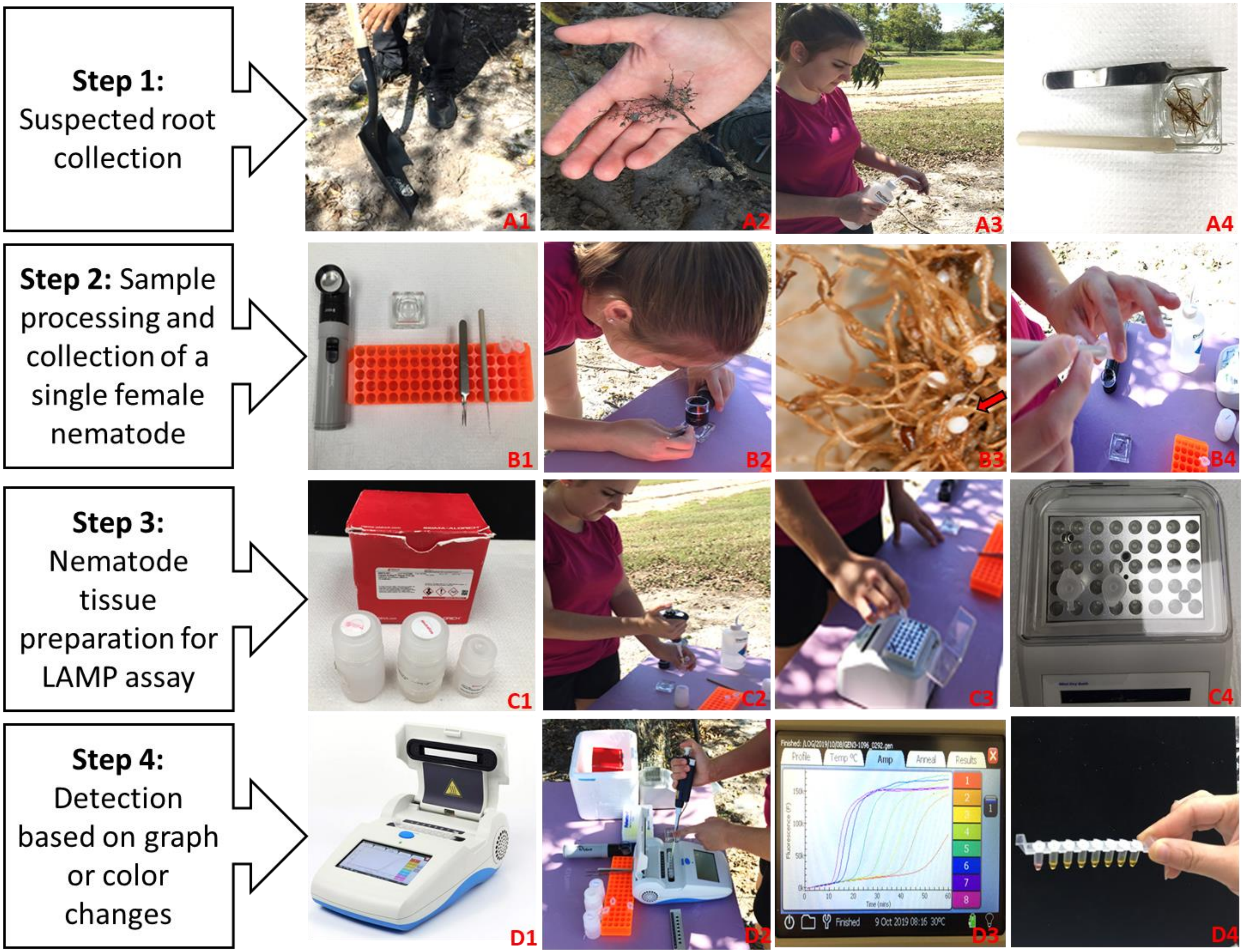

Figure S3 Layout for onsite diagnosis of *Meloidogyne partityla*
