# Supplementary material for: Rapid detection of pecan root-knot nematode, Meloidogyne partityla, in laboratory and field conditions using loop-mediated isothermal amplification
Source: PLoS One. 2020 Jun 18;15(6):e0228123. doi: 10.1371/journal.pone.0228123 (PMC7302683; doi:10.1371/journal.pone.0228123)

FIGURE 2 A) agarose gel electrophoresis of the LAMP products. Here, 1: 66ºC, 2: 67ºC, 3: 68ºC, 4: 69ºC, 5: 70ºC, 6: 71ºC, 7: 72ºC and 8: 73ºC. Lane M: 100 bp ladder marker.

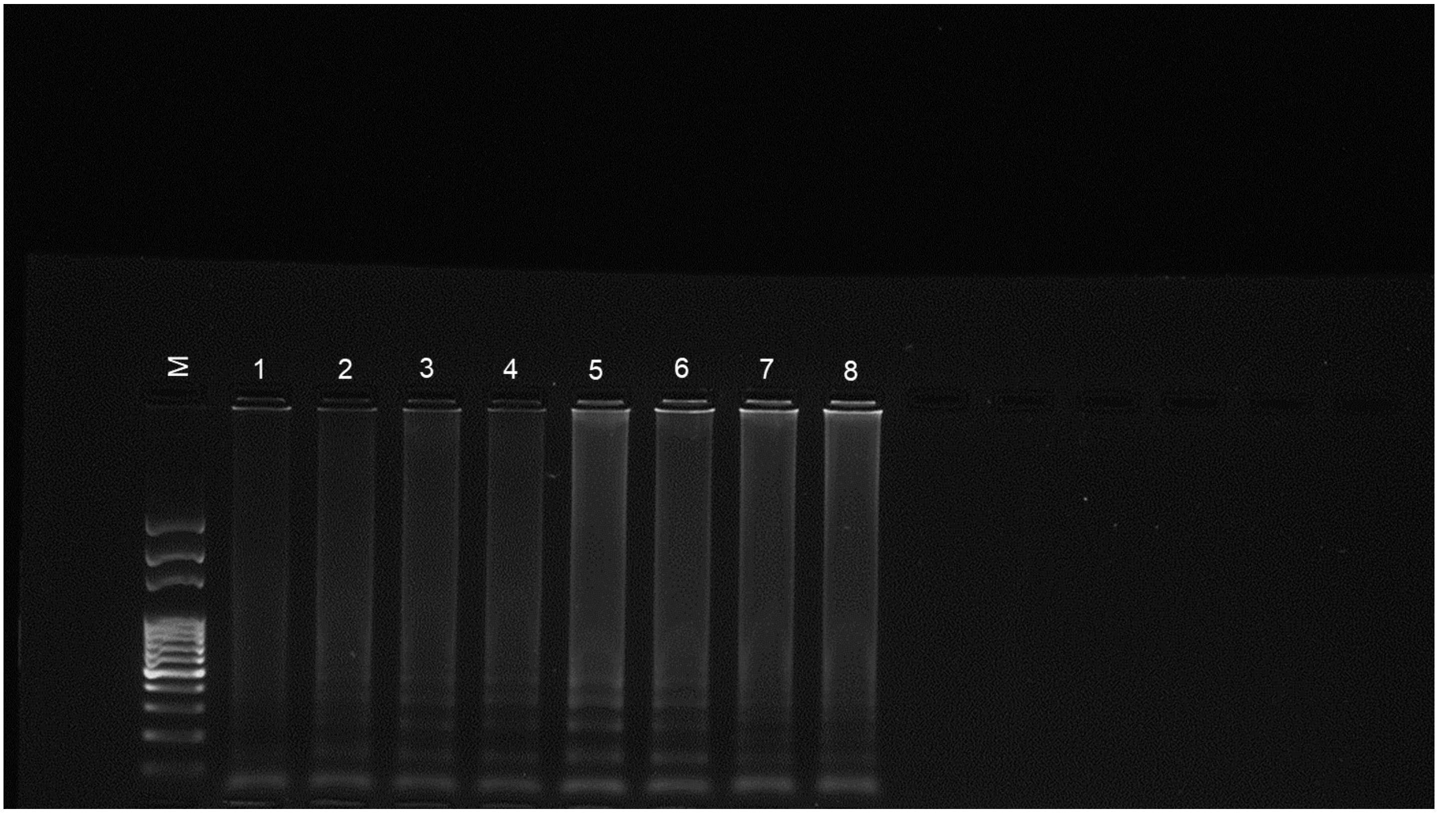

RIGHT-FIGURE 3 (A) agarose gel electrophoresis of the LAMP products. Here, lane M: 100 bp ladder marker; Mp1, Mp2, Mp3 and Mp4: four different isolates of Meloidogyne partityla; Neg: negative control.

LEFT-FIGURE 4 (A) agarose gel electrophoresis of the LAMP products. Here, lane M: 100 bp DNA ladder; M. part: Meloidogyne partityla; M. hap: Meloidogyne hapla; M. jav: Meloidogyne javanica; M. inc: Meloidogyne incognita; M. are: Meloidogyne arenaria; Neg: negative control.

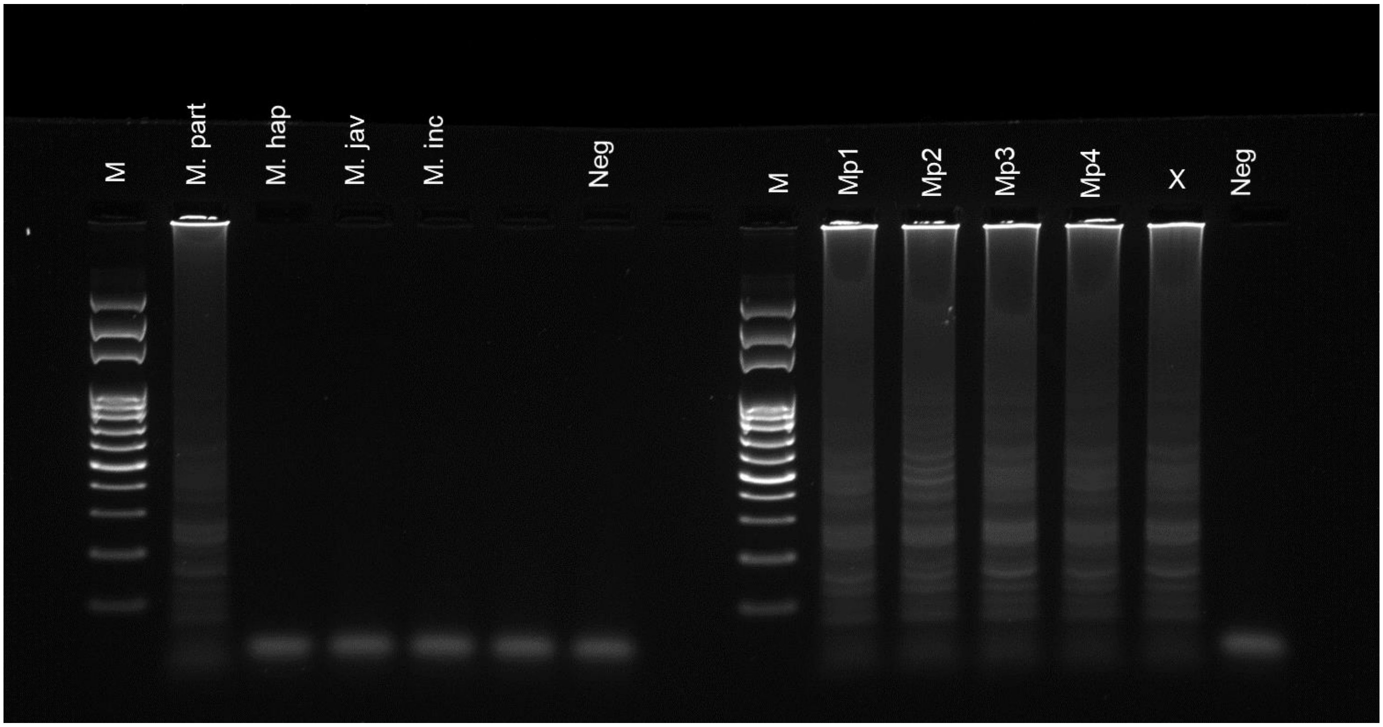

Figure 3 (D) PCR amplification using primer pair C2F3/1108. Lane M: 100 bp DNA ladder; Mp1, Mp2, Mp3 and Mp4: four different isolates of Meloidogyne partityla; Neg: negative control.

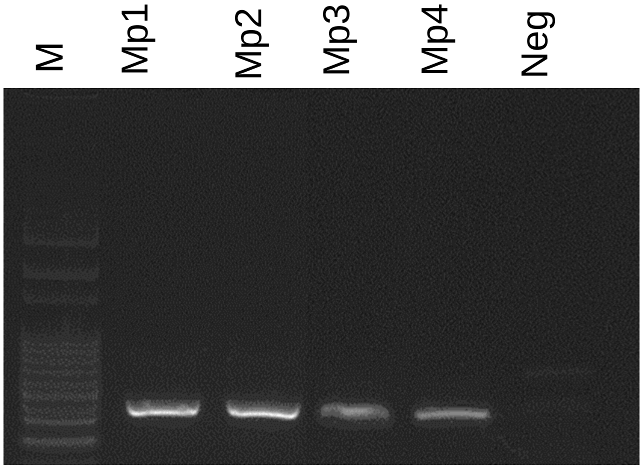

FIGURE 5 (A) agarose gel electrophoresis of the LAMP products. Lane M: 100 bp DNA ladder; numbers 1 to 7: 10-fold serial dilution of M. partityla DNA from 100 ng/µl to 10−4 ng/µl; Neg: negative control.

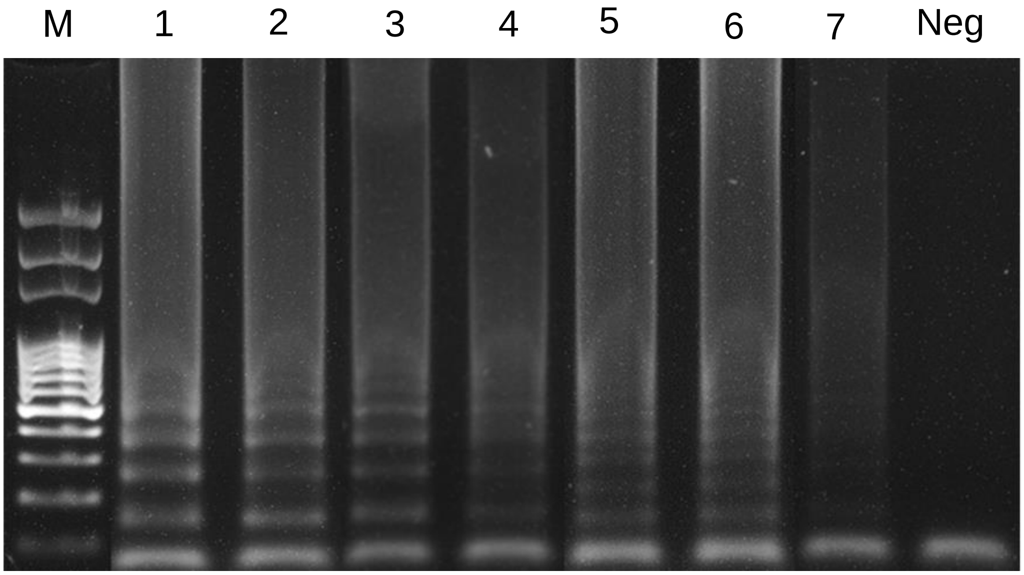

FIGURE 5 (D) PCR amplification using primer pair C2F3/1108. Lane M: 100 bp DNA ladder; numbers 1 to 7: 10-fold serial dilution of M. partityla DNA from 100 ng/µl to 10−4 ng/µl; Neg: negative control.

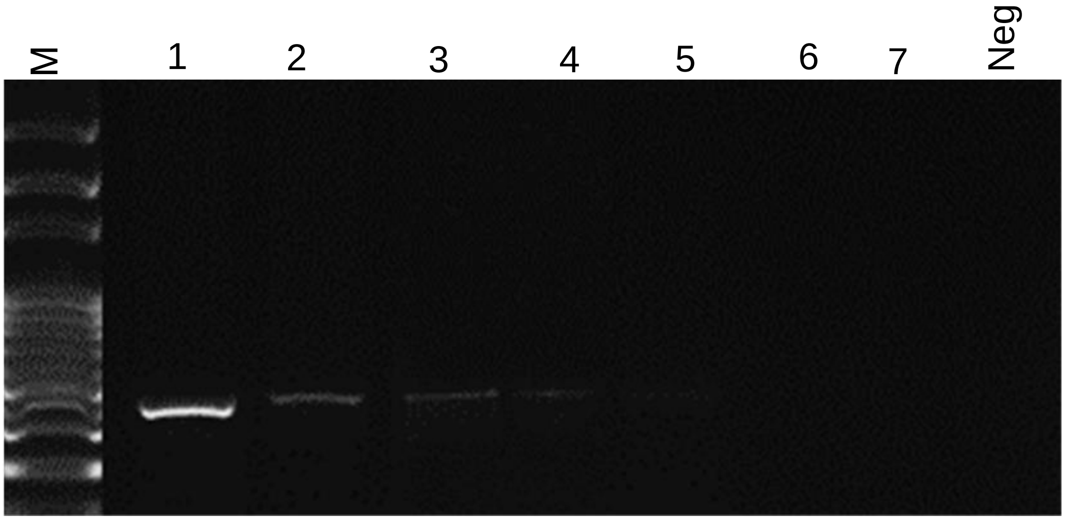

Supplement: S1 Raw images — (PDF) [file pone.0228123.s005.pdf]
